# Does digital infrastructure improve individuals’ body weight? A quasi-natural experiment based on “Broadband China” policy

**DOI:** 10.3389/fpubh.2026.1852852

**Published:** 2026-08-20

**Authors:** Hongli Zhao

**Affiliations:** School of Labor Economics, Capital University of Economics and Business, Beijing, China

**Keywords:** BMI, broadband policy, difference-in-differences, digital infrastructure, health inequality, obesity

## Abstract

Whether digital infrastructure development improves population health outcomes remains empirically contested. This study exploits China’s “Broadband China” pilot policy—implemented in staggered batches across 117 cities during 2014–2016—as a quasi-natural experiment and employs a difference-in-differences (DID) design with seven waves of the China Family Panel Studies (CFPS, 2010–2022) to estimate the causal effect of broadband infrastructure expansion on individual body weight and weight-related health inequality. The main findings are threefold. First, the “Broadband China” policy significantly reduces individual BMI by 0.254 units and lowers the BMI relative deprivation index by 0.004, indicating simultaneous improvements in average body weight and reductions in health inequality. Second, heterogeneity analyses reveal a dual-edged pattern: the policy disproportionately benefits vulnerable populations—including residents in economically underdeveloped regions, women, and older adults—thereby narrowing digital health divides, yet exerts stronger effects among highly educated groups, suggesting that educational disparities may perpetuate inequality through differential digital health literacy. Third, mechanism analysis reveals that the policy functions through four pathways: improving mental health, enhancing life satisfaction, cutting working hours and raising physical activity time. In terms of negative competitive mechanisms, the policy also exerts adverse effects by delaying sleep time and increasing sedentary duration. These findings provide policy-relevant evidence for leveraging digital infrastructure as a public health intervention tool in developing countries.

## Introduction

Health is regarded as one of the most important factors of social and economic development (1). For individuals, a healthy body is not only the foundation for enhancing human capital (2) and increasing income (3), but also a source of well-being (4). For society, improving public health can reduce medical expenditures and the social burden of healthcare, as well as boost labor productivity (5). As a good indicator of disease risk, body weight is frequently used to measure residents’ physical health, while the relative weight deprivation index can be employed to assess health inequality. As a populous country, China has witnessed a continuous rise in the average body mass index (BMI) and obesity rate among adults since the 1980s. According to the latest data from the *Report on Nutrition and Chronic Diseases of Chinese Residents (2020)*, more than half of Chinese adults are now overweight or obese. Therefore, improving the body weight and physical fitness of the population has become one of the important goals of the Chinese government.

Existing studies suggest that factors influencing body weight can be summarized into three categories. The first is innate factors, such as the impact of genetic endowment. The second is lifestyle habits; researchers have verified that unhealthy lifestyles, including a high-fat diet (6), alcohol consumption (7), physical inactivity (8), and insufficient sleep (9), can contribute to obesity. The third is socioeconomic and cultural factors, such as the relationship between social status and body weight, as well as that between educational attainment and body weight.

Nowadays, digitalization has become an irreversible trend, deeply permeating people’s daily lives and work. According to the *Statistical Report on Internet Development in China*, by 2025, the number of internet users in China had reached 1.125 billion, with an internet penetration rate of 80.1%. The number of generative AI users stood at 602 million, raising its penetration rate to 42.8%. Digital development is inseparable from the improvement of digital infrastructure. In 2013, the Chinese government launched the “Broadband China” Strategy, comprehensively promoting the construction and popularization of high-speed broadband networks. In 2014, the Ministry of Industry and Information Technology (MIIT) and the National Development and Reform Commission (NDRC) approved 39 pilot cities for the Broadband China initiative. Subsequently, the number was expanded to 81 cities in 2015 and a further 36 in 2016, bringing the total number of “Broadband China” Model Cities to 117. This policy has greatly propelled the development of China’s internet industry.

Evidence suggests that digitalization exerts a profound impact on numerous determinants of body weight, such as income distribution (10) and health-related behaviors (11, 12). Therefore, it is necessary to investigate: What effect does China’s implementation of the “Broadband China” Strategy have on body weight? Can the “Broadband China” Strategy mitigate the trend of health inequality? And what is the mechanism through which “Broadband China” operates?

To address the above questions, this study uses seven waves of CFPS data from 2010 to 2022 and adopts the DID method to explore the impact and mechanism of the “Broadband China” policy shock on Chinese residents’ body weight and weight gap, thereby providing Chinese empirical evidence for the relevant research field. The structure of this paper is as follows: the second part presents the literature review and hypothesis development; the third part describes the model specification and variable selection; the fourth part reports the results of the empirical analysis; and the fifth part draws conclusions and puts forward targeted policy recommendations.

## Literature review and hypothesis development

### The effect of digital infrastructure on body weight

This study draws on two competing theoretical perspectives—health promotion theory and technostress theory—to construct a unified analytical framework that generates opposing predictions regarding the impact of digital infrastructure on body weight.

According to health promotion theory (13) improvements in the information environment and resource accessibility empower individuals to make better health decisions (14, 15). Digital infrastructure expansion broadens access to health information, reduces barriers to online medical consultation, and facilitates the adoption of health-enhancing behaviors (16, 17). This perspective predicts that broadband penetration will reduce obesity through enhanced health literacy, improved mental health via expanded social support networks, and increased physical activity enabled by flexible work arrangements and online fitness resources (18).

In contrast, technostress theory (19) posits that intensive digital technology exposure generates psychological and behavioral burdens. Broadband expansion may increase sedentary screen time, disrupt sleep patterns, induce information overload and compulsive connectivity, and facilitate access to calorie-dense delivered food—all of which elevate obesity risk (20–23).

These two theoretical perspectives generate competing predictions that cannot be resolved *a priori*. The net effect of digital infrastructure on body weight is an empirical question determined by the relative strength of health-promoting versus health-undermining channels. This study leverages the “Broadband China” quasi-natural experiment to empirically adjudicate between these competing predictions while simultaneously examining the heterogeneity of net effects across population subgroups.

Currently, there is relatively limited discussion on the relationship between digitalization and weight inequality. On the one hand, due to the existence of the digital divide, the transmission of digital inequality into the health domain may exacerbate weight inequality. On the other hand, the widespread diffusion of the internet has made access to health information and resources more convenient (24), greatly improving the accessibility of medical resources, which is particularly beneficial to people in remote areas, busy individuals, and vulnerable groups (25). Meanwhile, healthcare providers can leverage the internet to upgrade their professional skills and knowledge, offering assistance and guidance to patients when needed (26, 27). The “Broadband China” policy implemented by the Chinese government has effectively narrowed the digital access divide and promoted the flow of medical resources to remote regions and vulnerable groups beyond temporal and spatial constraints, thereby effectively alleviating weight inequality caused by unequal distribution of medical resources.

### Mechanisms of digital infrastructure on body weight

#### Digital infrastructure and mental health improvement

From a psychological perspective, negative emotions such as stress, anxiety, and depression can increase the risk of obesity (28, 29). The development of digital infrastructure reduces the perceived spatial distance in social networks and strengthens the intensity of interpersonal interactions (30). On one hand, Cobb (31) noted that the development of the Internet has exerted a significant “buffering effect” on individuals’ physical and mental health. Digital infrastructure expands users’ social networks, providing individuals with social opportunities and channels for emotional expression. Research indicates that social capital and social support contribute to improved physical and mental health (32, 33). Broadband penetration reduces the cost of information access, enabling residents to obtain information on education, healthcare, and employment more easily. This indirectly improves life convenience and sense of gain, thereby enhancing life satisfaction. Meanwhile, digital infrastructure promotes the development of online public services, allowing residents in remote areas to access public services equitably and improving their livelihood security and life satisfaction.

#### Digital infrastructure and the reshaping of daily habits

From the perspective of lifestyle habits, prolonged sitting, reduced physical activity, and reliance on transportation for commuting all increase the likelihood of obesity (34). Conversely, reducing sedentary time and increasing exercise frequency decrease the likelihood of obesity (35). On the one hand, as work becomes increasingly mechanized and automated, people have gradually adopted a sedentary lifestyle, which raises the risk of obesity. The implementation of the “Broadband China” strategy has promoted the popularization of diverse work models such as telecommuting and flexible working systems, effectively reducing the total actual working hours of employees (36–38). Individuals can utilize more leisure time to improve their lifestyle, dietary habits, and weight management. On the other hand, the internet has eliminated time and space constraints, enabling employees to exercise during office breaks (39). Moreover, a large number of low-cost or free online resources are available, enabling people to exercise at a relatively low cost (40). Some scholars also note that the internet accelerates the spread of fitness trends. During the COVID-19 pandemic, the Chinese celebrity Liu Geng Hong shared fitness tips and routines via live streaming platforms, inspiring many people to follow his fitness programs and improving their body weight conditions (41).

#### Competitive mechanism: potential negative transmission pathways

The above discussions elaborate the health-enhancing potential of digital infrastructure from the perspective of health promotion. Nevertheless, technology stress theory indicates that broadband expansion may also function through negative pathways that raise obesity risks.

The first competitive pathway is psychological overload. Widespread digital connectivity tends to result in information overload, compulsive engagement in online entertainment and constant connectivity pressure (42). Long-term digital immersion can induce cognitive fatigue, sleep disturbances and sustained psychological stress. The accumulated negative emotions adversely affect individuals’ sleep duration and quality (43), and further increase obesity risks via pathways that counteract the positive effects of mental health improvement.

The second competitive pathway manifests as the consolidation of sedentary behaviors. Digital infrastructure substantially lowers the participation cost of screen-based sedentary activities, including online games, video streaming and social media browsing. It accordingly extends daily screen time, entrenches sedentary lifestyles and squeezes out time allocated to outdoor physical activities (34). Such sedentary behavior acts as a competitive channel that partially offsets the positive lifestyle restructuring effects brought by broadband popularization (44).

Accordingly, the net impact of digital infrastructure on body weight remains an empirical question, which hinges on the relative strength of health-promoting and health-harming channels operating simultaneously within specific institutional and behavioral contexts.

#### Research review and hypothesis development

Existing studies have covered digitalization and health, digitalization and health inequality, and the mechanisms through which digitalization affects health. These studies exhibit the following three characteristics. First, discussions of digitalization mainly focus on the usage side, examining how internet use, screen time, and similar factors influence health, while insufficient attention has been paid to the supply-side impact of digital infrastructure on residents’ health. Digital infrastructure constitutes the foundation of digital development and technology application and reflects policy input from the government. Investigating its effect on residents’ health enables targeted evaluation of government policies and helps formulate more constructive recommendations. Second, most studies adopt subjective health measures, such as self-rated health scores. However, self-rated health is heavily affected by individual perceptions and may not fully and accurately reflect actual health status. In contrast, BMI, a physical fitness index calculated from height and weight, can objectively measure obesity and overweight conditions and reasonably represent individual health levels. Third, relevant studies have not yet reached a consistent conclusion. Moreover, given that China is in a period of economic and social transition, it remains unclear whether existing international findings apply to the Chinese context.

Therefore, this paper uses seven waves of CFPS data from 2010 to 2022 and employs the difference-in-differences (DID) method to examine the effects and mechanisms of the “Broadband China” policy shock on residents’ body weight and weight disparity, providing empirical evidence from China for related research fields.

Based on the above analysis, this paper proposes the following four hypotheses:

H1: Digital infrastructure construction can effectively reduce obesity and improve health inequality.

H2: Digital infrastructure construction can improve individual mental health, thereby reducing obesity and alleviating health inequality.

H3: Digital infrastructure construction can reshape individual lifestyle habits, thereby curbing obesity and mitigating health inequality.

H4: Digital infrastructure may generate adverse psychological effects and reinforce sedentary behaviors simultaneously, thereby partially offsetting the health benefits identified in Hypotheses H1 to H3.

## Materials and methods

### Model design

The difference-in-differences (DID) method is an econometric approach widely used in recent years to evaluate the effects of policy implementation. This method treats the implementation of government policies as a quasi-natural experiment outside the economic system, thereby assessing the economic and social impacts of pilot policies. The implementation of the “Broadband China” pilot policy not only creates differences in residents’ physical health status before and after the policy implementation within the same pilot city but also may have different impacts on the physical health of residents in pilot cities and non-pilot cities at the same point in time. Therefore, this study employs an overlapping difference-in-differences (ODID) model to estimate and identify the net impact of this policy on residents’ physical health, and constructs the following model:

$${\text{Agency}}_{\mathrm{ip}}=\alpha_{0}+\alpha_{1}{\text{Broadband}}_{\mathrm{ip}}+\alpha_{2}CV_{\mathrm{ip}}+\delta_{i}+\eta_{p}+\mu_{\mathrm{ip}}$$ (1)

In Equation (1), the dependent variable ${\text{Agency}}_{\mathrm{ip}}$ represents the body weight of individual *i* in year *p*. The core explanatory variable ${\text{Broadband}}_{\mathrm{ip}}$ indicates whether the city where individual *i* resides implemented the “Broadband China” pilot policy in year *p*. It takes a value of 1 if the pilot policy was implemented and 0 otherwise. $\delta_{i}$ represents the city fixed effect, $\eta_{p}$ denotes the time fixed effect, and $\mu_{\mathrm{ip}}$ represents the random error term. $\alpha_{1}$ is the difference-in-differences estimator, measuring the impact of the “Broadband China” pilot policy on residents’ physical condition at the individual micro level. This is the primary focus of estimation in this paper. ${\mathrm{CV}}_{\mathrm{ip}}$ represents a set of control variables affecting the physical condition of individual *i* in year *p*.

### Variable selection

#### Dependent variable

According to the National Health and Family Planning Commission of the People’s Republic of China (2013), obesity can be measured using the Body Mass Index (BMI, kg/m^2^). Therefore, this study employs BMI to represent individual body weight. A higher BMI value indicates a greater likelihood of overweight or obesity, as well as a higher risk of poor physical health. The specific formula is as follows [see Equation (2)]:

$$\mathrm{BMI}=\frac{\text{weight}}{{\text{height}}^{2}}$$ (2)

To minimize the impact of severely obese individuals on the analysis results, the BMI values were truncated.

The theoretical rationale for employing the BMI relative deprivation index as a proxy for health inequality rests upon two considerations. First, BMI constitutes an objective, universally standardized biomarker that directly reflects nutritional status and adiposity while serving as a validated predictor of hypertension, type 2 diabetes, and cardiovascular disease (45). Unlike subjective self-rated health, which is susceptible to reporting bias and exhibits limited cross-group comparability, BMI provides a reliable and comparable foundation for inequality measurement. Second, within-group BMI differentials are not purely biological in nature but systematically embed inequalities in socioeconomic resources—including income, educational attainment, health information accessibility, dietary quality, and public health service allocation—that cumulatively shape individual weight outcomes. The relative widening of BMI gaps across population subgroups therefore reflects structurally determined disparities in the social determinants of health (46, 47), rendering the BMI relative deprivation index a valid proxy for health inequality rather than merely a measure of biological variation.

To construct the BMI relative deprivation index, we first categorize respondents by BMI level and assign corresponding health scores: normal weight (18.5 ≤ BMI < 24) receives a score of 3, representing the health-optimal range; underweight (BMI < 18.5) and overweight (24 ≤ BMI < 28) each receive a score of 2, reflecting moderate deviations from the optimal range that carry elevated health risks including malnutrition, osteoporosis, and compromised immune function for the former, and increased cardiovascular risk for the latter; and obese (BMI ≥ 28) receives a score of 1, indicating the most severe deviation associated with the highest disease burden. This non-monotonic scoring scheme explicitly acknowledges that health risks are bidirectional—both insufficient and excessive body weight deviate from the optimal state—while preserving the distinction between moderate and severe health risk levels. Second, let *X* be a sample set consisting of n individuals. The individuals are sorted by body weight score from lowest to highest, yielding *X* = ( $x_{1},x_{2},\ldots ,x_{n}$ ), where $x_{1}$ ≤ $x_{2}$ ≤ … $x_{n}$ . By comparing the body scores of individual *i* and individual *j*, the degree of relative deprivation in body composition for individual i can be obtained [see Equation (3)]:

$$\begin{array}{c} \mathrm{RD}(x_{j},x_{i})=\{\begin{array}{c} x_{j}-x_{i}\;\;\text{if}\;\;x_{j}>x_{i}\\ 0\;\;\;\text{if}\;\;\;x_{j}\leq x_{i} \end{array} \end{array}$$ (3)

Compare individual i’s physique score with those of all other individuals and calculate their average relative deprivation in BMI [see Equation (4)]:

$$\begin{array}{c} \mathrm{RD}(x_{i})=\frac{1}{n\mu_{x}}(n_{\mathrm{xi}}^{+}\times n_{\mathrm{xi}}^{+}-n_{\mathrm{xi}}^{+}\times x_{i})=\frac{1}{\mu_{x}}\gamma_{\mathrm{xi}}^{+}(n_{\mathrm{xi}}^{+}-x_{i}) \end{array}$$ (4)

Here, *μₓ* represents the average body weight score of the entire sample; $n_{\mathrm{xi}}^{+}$ denotes the number of individuals with a body weight score higher than that of individual *i*; $n_{\mathrm{xi}}^{+}$ denotes the average body weight score of the group of individuals with a body weight score higher than that of individual i; and $\gamma_{\mathrm{xi}}^{+}$ denotes the proportion of individuals with a body weight score higher than $x_{i}$ .

The BMI relative deprivation index reflects an individual’s relative position within the group, taking into account the comparison of the individual’s body weight with that of all other members. A higher index indicates that the individual’s body weight is relatively less favorable compared to other members of the group, suggesting a greater relative disparity in factors related to health and quality of life.

#### Explanatory variable

Drawing on the research of Niu and Cui (48), this paper uses the implementation of the “Broadband China” policy as a proxy variable for digital infrastructure development. If an individual’s city is included in the pilot or expansion cities of the “Broadband China” initiative and is located after the policy’s commencement, this variable is assigned a value of 1; otherwise, it is assigned a value of 0.

#### Control variables

This study references existing literature (49, 50) and incorporates the following control variables: age, age squared, gender (male = 1, female = 0), educational attainment (illiterate/semi-literate = 1, elementary school = 2, junior high school = 3, senior high school/vocational school/technical school/vocational high school = 4, junior college = 5, bachelor’s degree and above = 6), marital status (married = 1, unmarried = 0), household registration status (urban = 1, rural = 0), smoking status, drinking status, and presence of chronic disease.

#### Instrumental variables

This paper adopts the methodology of Fang et al. (51), selecting the distance between each city and Hangzhou as an instrumental variable for the “Broadband China” policy. Hangzhou is the birthplace and core hub of China’s digital economy; therefore, the closer a city is to Hangzhou, the more likely it is to possess the foundation for digital infrastructure development and application, and thus the more likely it is to become a pilot site for the “Broadband China” policy. This distance indicator is significantly correlated with the selection of policy pilot sites. As a historically formed geographic variable with strong exogeneity, it satisfies the identification assumptions for an instrumental variable.

#### Mechanism variable

This paper examines the mechanisms through which digital infrastructure impacts residents’ physical health from two dimensions: enhancing mental health and reshaping lifestyles.

Regarding the mechanisms underlying improvements in mental health, this study selected mental health and life satisfaction as the two outcome variables. For “mental health,” the China Family Panel Studies (CFPS) used the K6 scale in 2010 and 2014, the CESD-20 scale in 2012 and 2016, and the CESD-8 scale in 2018, 2020, and 2022 to assess mental health status. Therefore, following the methodology of Liu et al. (52), this study standardized the total scores of all items on the K6 and CESD scales for the period from 2010 to 2022. Higher scores indicate poorer mental health. Regarding “life satisfaction,” CFPS respondents answered questions regarding “their satisfaction with their lives.” In this study, responses ranging from “very dissatisfied” to “very satisfied” were assigned values from 1 to 5, with higher values indicating greater satisfaction.

Regarding the mechanisms of lifestyle reshaping, this paper selected two variables—working hours and exercise duration—for measurement. For “working hours,” the study utilized a question from the China Family Panel Studies (CFPS) questionnaire: “Including overtime hours but excluding lunch breaks, regardless of whether paid or unpaid, what were your daily working hours (in hours) over the past 12 months?” Regarding “exercise duration,” this paper utilizes the question from the China Family Panel Studies (CFPS) questionnaire: “Over the past 12 months, how many hours did you spend on average per exercise session?”

We select sleep time and sedentary duration to examine the competitive mechanism. Sleep time delay is measured by the question regarding respondents’ usual bedtime at night in the CFPS data, while daily internet usage time from CFPS is used to represent sedentary duration.

### Data source

The data used in this study are from the China Family Panel Studies (CFPS) database. Conducted by the Institute of Social Science Survey (ISSS) of Peking University, this database is a longitudinal tracking survey covering 25 provinces in China and featuring national representativeness. This study uses seven waves of publicly available data from 2010 to 2022 as the research sample. Data on the pilot cities and policy timeline of the “Broadband China” program are sourced from the Ministry of Industry and Information Technology of the People’s Republic of China. City-level macro data are obtained from the *China City Statistical Yearbook.* First, this study matches the CFPS data from 2010 to 2022 with the list of pilot cities of the “Broadband China” policy and household income data at the prefecture-level city level. Subsequently, it cross-compares the matched data with other city-level data, and finally obtains a total of 171,051 research observations. Table 1 presents the basic information for each variable.

**Table 1:** Descriptive statistics.

Variable	The entire sample		Treatment group		Control Group	
	*N* = 171,051		*N* = 57,116		*N* = 113,122	
	Mean	Std.	Mean	Std.	Mean	Std.
BMI	22.65	3.43	22.93	3.37	22.50	3.45
BMI-RD	0.13	0.09	0.13	0.09	0.12	0.09
Policy × year	0.17	0.38	0.26	0.44	0.13	0.33
Age	46.04	16.58	47.19	16.74	45.45	16.48
Gender	0.50	0.50	0.49	0.50	0.50	0.50
Edu	2.69	1.35	2.98	1.39	2.55	1.30
Marry	0.79	0.41	0.80	0.40	0.79	0.41
Hukou	0.71	0.45	0.58	0.49	0.78	0.42
Inincome_w	4.24	4.76	4.93	4.90	3.89	4.64
Smoke	0.29	0.45	0.29	0.45	0.29	0.45
Drink	0.15	0.35	0.16	0.36	0.14	0.35
Chronic	0.15	0.36	0.17	0.37	0.15	0.36

## Empirical results

### Baseline analysis

Table 2 reports the estimates of the impact of digital infrastructure development on body weight from the difference-in-differences model. Columns (1) and (2) present the results for BMI and the BMI relative deprivation index, respectively; all models control for fixed effects of city and year. Due to space constraints, subsequent tables will not include the estimates of the control variables. The double-difference estimation results indicate that, for both the regression results on BMI and BMI relative deprivation index, the interaction term coefficients are significantly negative at the 1% statistical level. After the implementation of the pilot policy, the treatment group experienced significant declines of 0.254 and 0.004 in the two indicators relative to the control group. Such decreases correspond to relative reductions of 1.12 and 3.08% against the sample mean, which are modest yet non-negligible at the population level. If this effect persists stably, it can substantially reduce the burden of cardiovascular and metabolic diseases as well as alleviate health inequality nationwide. Accordingly, Hypothesis 1 is verified.

**Table 2:** Regression results.

Variables	BMI	BMI-RD
	(1)	(2)
Broadband	−0.254***	−0.004***
	(0.032)	(0.001)
Age	0.230***	0.002***
	(0.003)	0.000
Age^2^	−0.002***	−0.000***
	0.000	0.000
Gender		
Men	0.779***	0.002***
	(0.020)	(0.001)
Edu		
Primary	0.162***	0.005***
	(0.025)	(0.001)
Junior secondary	0.075***	0.007***
	(0.025)	(0.001)
Senior secondary	−0.061**	0.008***
	(0.029)	(0.001)
College	−0.157***	0.008***
	(0.041)	(0.001)
University	−0.411***	0.009***
	(0.047)	(0.001)
Marry		
Married	0.609***	0.005***
	(0.024)	(0.001)
Setting		
Urban	−0.575***	0.002***
	(0.022)	(0.001)
Income	0.004**	0.000***
	(0.002)	0.000
Smoke	−0.409***	0.001
	(0.022)	(0.001)
Drink	0.053**	0.001
	(0.024)	(0.001)
Chronic	0.295***	−0.007***
	(0.023)	(0.001)
Constant	17.361***	0.071***
	(0.068)	(0.002)
City fixed effect	Yes	Yes
Time fixed effect	Yes	Yes
*R* ^2^	0.153	0.024
Observations	171,051	171,051

***, **, and * in the table denote significance levels at 1, 5, and 10%, respectively, and standard deviations are in parentheses, as below.

### Robustness test

#### Endogenous test

To address potential endogeneity issues including reverse causality, omitted variable bias and measurement error, this study adopts the geographical distance from each city to Hangzhou as the instrumental variable for the Broadband China policy (51). Column (1) in Table 3 presents the first-stage regression results. In terms of correlation, as a core birthplace of China’s digital economy and digital inclusive finance, Hangzhou features geographically decaying diffusion of technology and business. Shorter geographical distance corresponds to higher penetration of digital industries. The instrumental variable is significantly negatively correlated with the core explanatory variable, and the *F*-statistic far exceeds the Stock–Yogo critical value, ruling out the weak instrumental variable problem. In terms of exogeneity, the distance to Hangzhou is determined by longitude and latitude as a purely natural geographic factor, which is unaffected by economic, policy and social changes. Columns (2) and (3) report the estimation results of two-stage least squares regression, which are consistent with the baseline findings.

**Table 3:** Endogenous test results.

Variables	Broadband	BMI	BMI-RD
	(1)	(2)	(3)
Broadband		−0.831^***^	−0.019^***^
		(0.275)	(0.007)
Indistance_hangzhou	−0.081***		
	(0.002)		
Constant	0.401***	17.827***	0.791***
	(0.018)	(0.145)	(0.003)
Province fixed effect	Yes	Yes	Yes
Time fixed effect	Yes	Yes	Yes
*R* ^2^		0.135	0.016
Observations		171,051	171,051
Kleibergen–Paap rk LM statistic	880.25		
Kleibergen–Paap rk Wald *F* statistic	1403.717		
Stock–Yogo weak ID test critical values	16.38		
10% maximal IV size			

***, **, and * in the table denote significance levels at 1, 5, and 10%, respectively, and standard deviations are in parentheses.

Nevertheless, this instrumental variable has certain exogeneity defects and may be confounded by regional characteristics. To mitigate endogeneity risks arising from imperfect exclusion restriction, this study conducts three robustness treatments. First, regional fixed effects are incorporated to eliminate the confounding influence of regional economic development channels. Second, individual-level covariates such as income, education, household registration and chronic diseases are already controlled in baseline regressions to block alternative transmission paths from geographical distance to residents’ body weight via socioeconomic attributes. Third, this study employs the Union of Confidence Intervals method proposed by Conley et al. (53) to permit reasonable direct effects of instrumental variables and verify the validity of core conclusions. Following prevailing empirical practices, this paper sets 0.5 and 10 times the baseline coefficient as the lower and upper bounds of plausible direct impacts. The results show that the 95% confidence intervals of core coefficients are [−650.83, −35.14] and [−595.25, −12.53] respectively (Table 4). Both intervals remain significantly negative without crossing zero. It confirms that even allowing for moderate endogenous bias of the instrumental variable, the core conclusion that the Broadband China policy significantly reduces residents’ BMI and health inequality remains robust and reliable.

**Table 4:** Robustness check for instrumental variable estimation.

Variable		Lower bound (1)	Upper bound (2)
BMI	did_kd	−650.83	−35.14
	_cons	27.87	117.99
BMIRD	did_kd	−595.25	−12.53
	_cons	1.43	85.85
Observations		170,184	170,184

#### Parallel trends test

**Figure 1:**
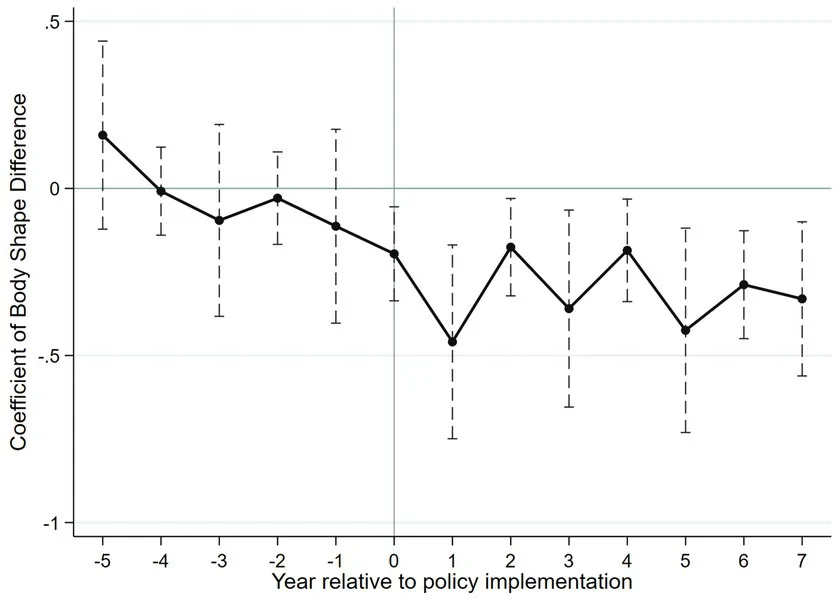
Parallel trend (BMI).

**Figure 2:**
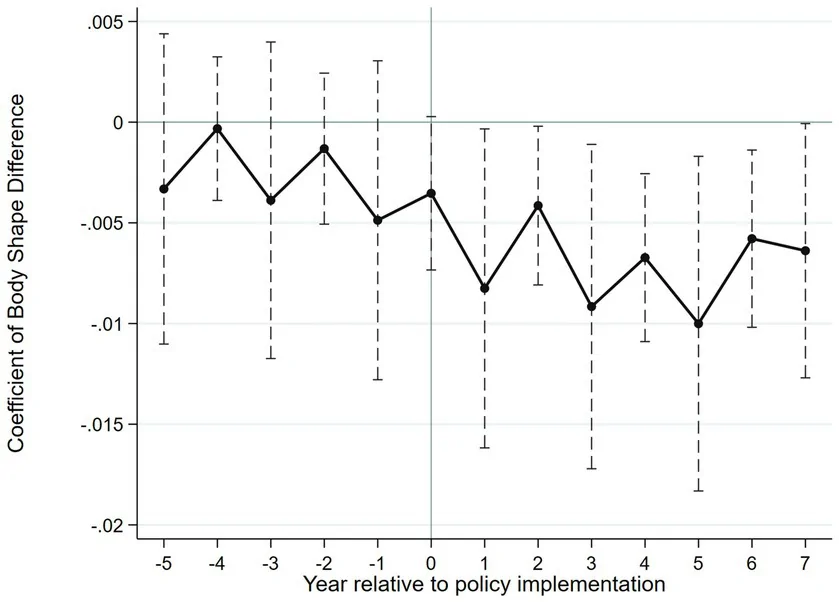
Parallel trend (BMI-RD).

The parallel trend test aims to confirm that no significant differences existed in individuals’ body weight between pilot cities and non-pilot cities prior to implementing the “Broadband China” policy. Figures 1, 2 show the parallel trends of BMI and BMI-RD. Compared to the baseline year, when the policy implementation time <0, the estimated coefficients, indicating no significant differences in body weight between residents of pilot cities and non-pilot cities.

#### Placebo test

**Figure 3:**
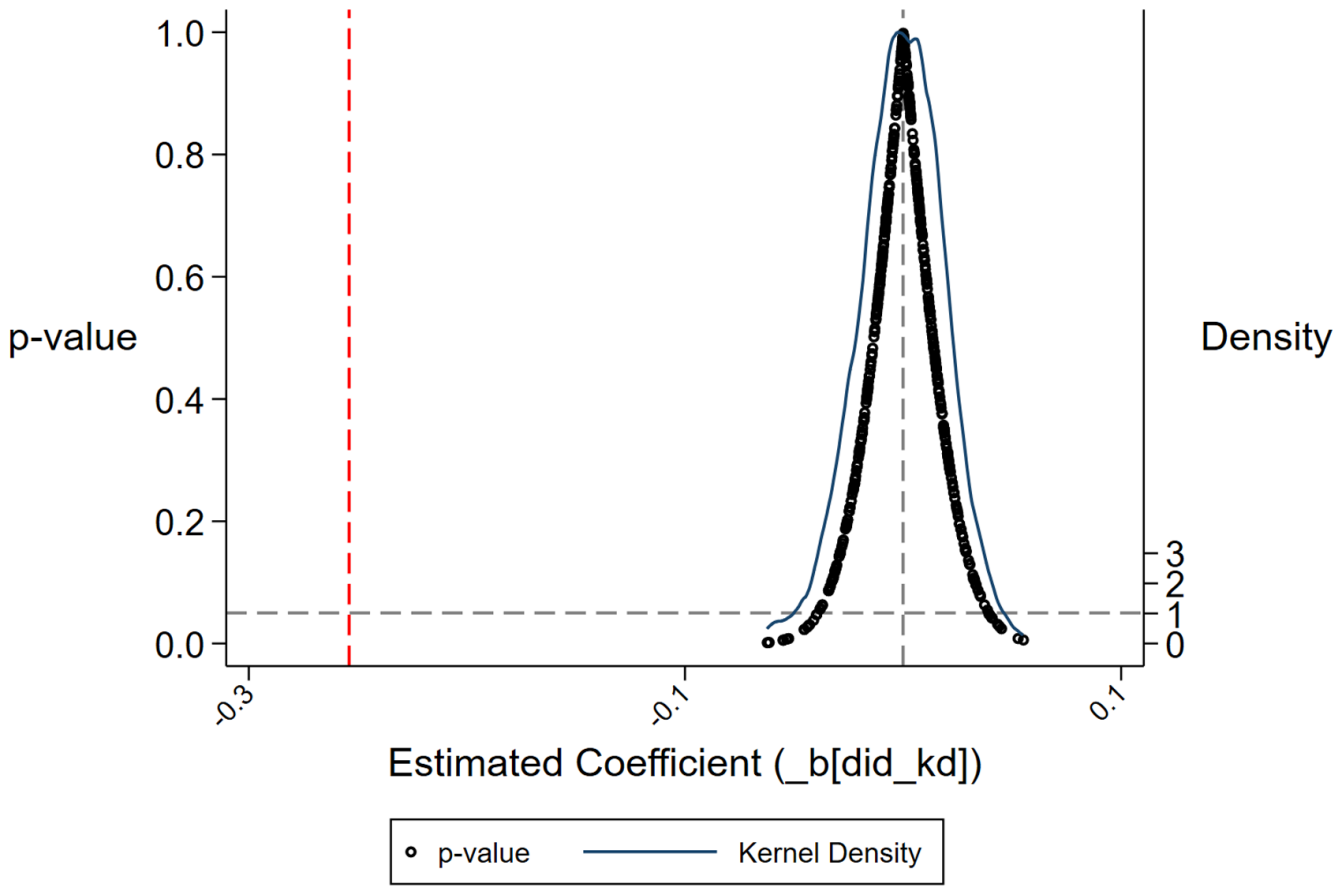
Placebo test (BMI).

**Figure 4:**
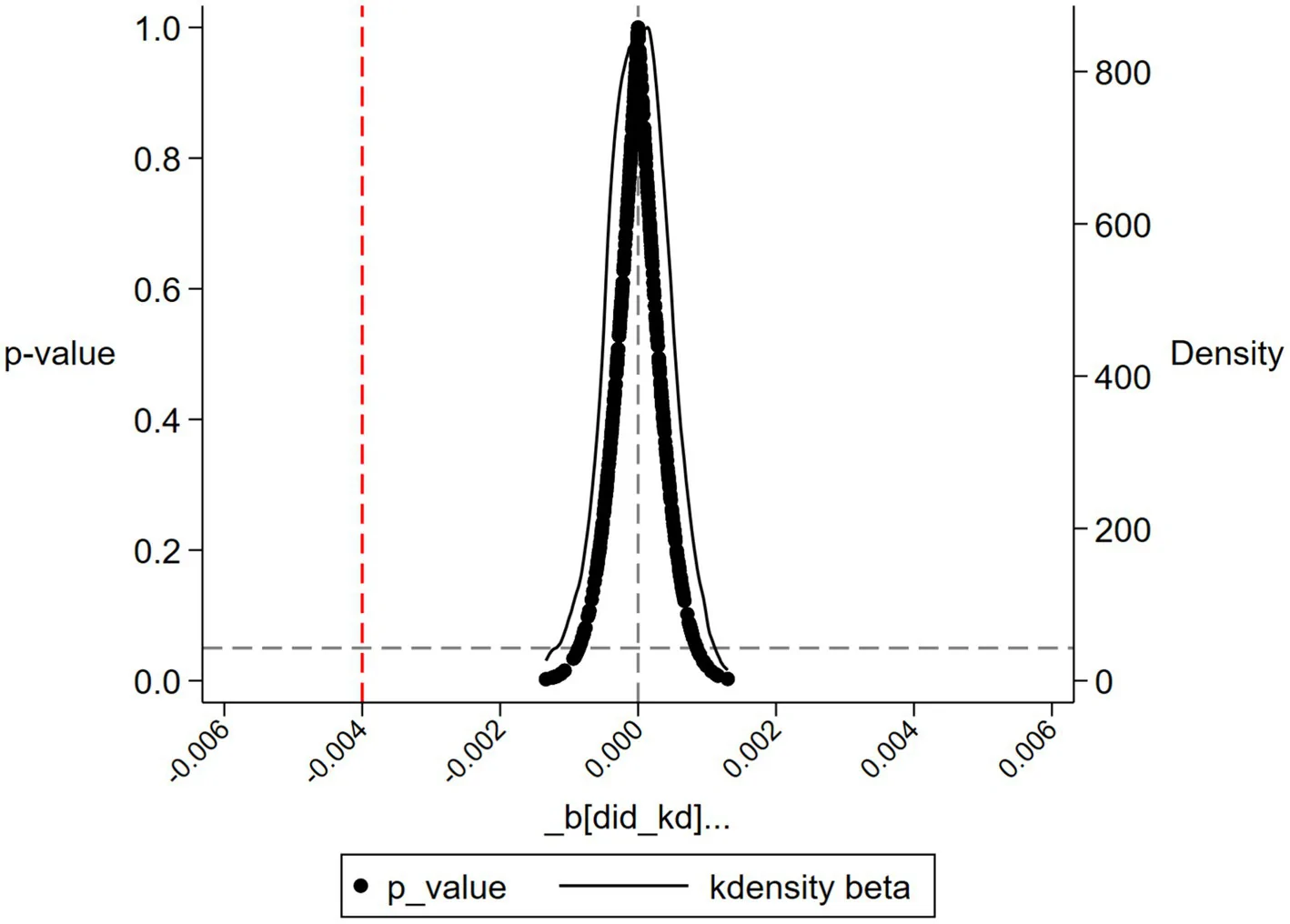
Placebo test (BMI-RD).

This study employed a random sampling method for placebo testing with a sampling frequency of 500 trials. Figures 3, 4 show the results of the kernel density distribution. As depicted, all *p*-values above the horizontal dashed line are non-significant, indicating that most simulated estimates are statistically insignificant. In contrast, the true estimate (red dashed line) lies at the far left of the distribution, distinctly different from the random results. This demonstrates the robustness of the benchmark regression conclusions, ruling out interference from random factors or omitted variables.

#### PSM-DID

In addition, we employ a propensity score-matched difference-in-differences (PSM-DID) model to address the endogeneity issues caused by sample selection bias. Given that the pilot cities for the “Broadband China” initiative were selected in phases, this study uses kernel matching, radius matching, and nearest-neighbor matching methods to pair the 117 pilot cities (treatment group) with corresponding non-pilot cities (control group) on an annual basis. Columns (1) and (2) of Table 5 report the DID estimates obtained through kernel matching. The results indicate that after eliminating sample selection bias via propensity score matching, our findings are consistent with those of the baseline regression. Furthermore, the DID estimates in Columns (3) through (6), obtained using radius matching and 1:1 non-replacement nearest-neighbor matching, respectively, yield identical conclusions.

**Table 5:** PSM-DID estimation results.

Variables	*K*-means		Radius matching		Nearest neighbor	
	BMI	BMI-RD	BMI	BMI-RD	BMI	BMI-RD
	(1)	(2)	(3)	(4)	(5)	(6)
Broadband	−0.358***	−0.006***	−0.358***	−0.006***	−0.350***	−0.010***
	(0.054)	(0.001)	(0.054)	(0.001)	(0.066)	(0.002)
Control Variable	Yes	Yes	Yes	Yes	Yes	Yes
	Yes	Yes	Yes	Yes	Yes	Yes
Constant	17.145***	0.077***	17.145***	0.077***	16.996***	0.077***
	(0.114)	(0.003)	(0.114)	(0.003)	(0.144)	(0.004)
City fixed effect	Yes	Yes	Yes	Yes	Yes	Yes
Time fixed effect	Yes	Yes	Yes	Yes	Yes	Yes
*R* ^2^	0.171	0.031	0.171	0.031	0.177	0.033
Observations	64,703	64,703	64,703	64,703	40,523	40,523

***, **, and * in the table denote significance levels at 1, 5, and 10%, respectively, and standard deviations are in parentheses.

#### Exclude the four municipalities directly under the central government

Considering that the four municipalities (Beijing, Shanghai, Tianjin, and Chongqing) enjoy distinct advantages in economic development, digital infrastructure, and exercise atmosphere, we exclude these cities to mitigate estimation bias and re-estimate our baseline model. Columns (1) and (2) in Table 6 present the results of this robustness test. These results are consistent with those of the baseline study.

**Table 6:** Results of robustness tests after controlling for centrally administered municipalities and contemporaneous policies.

Variables	BMI	BMI-RD	BMI	BMI-RD
	(1)	(2)	(3)	(4)
Broadband	−0.291***	−0.004***	−0.191***	−0.004***
	(0.033)	(0.001)	(0.034)	(0.001)
National high-tech zone policy	–	–	0.021	0
	–	–	(0.042)	(0.001)
Information-for-public-benefit policy	–	–	−0.211***	−0.001
	–	–	(0.036)	(0.001)
Control variable	Yes	Yes	Yes	Yes
	Yes	Yes	Yes	Yes
Constant	17.303***	0.069***	17.406***	0.071***
	(0.071)	(0.002)	(0.072)	(0.002)
City fixed effect	Yes	Yes	Yes	Yes
Time fixed effect	Yes	Yes	Yes	Yes
*R* ^2^	0.157	0.025	0.152	0.023
Observations	155,326	155,326	155,651	155,651

***, **, and * in the table denote significance levels at 1, 5, and 10%, respectively, and standard deviations are in parentheses.

#### Controlling synchronization policy

As the “National High-Tech Zone Policy” and “Information-for-Public-Benefit Policy” advance in tandem, these policies may also influence the relationship between regional digital infrastructure development and individual weight. Columns (3) and (4) in Table 6 show that even after controlling for the effects of “National High-Tech Zone Policies” and “Public Information Policies,” the results remain consistent with those of the baseline regression analysis. This indicates that the impact of other policies not included in the analysis on the baseline regression results is negligible.

### Heterogeneity analysis

Table 7 presents the regression estimates of the impact of the “Broadband China” pilot policy on body weight, taking into account factors such as economic development, urban–rural status, region, gender, age, and educational attainment. Heterogeneity analysis indicates that, on the one hand, the policy effectively bridges the digital health divide that may result from uneven coverage of digital infrastructure. Heterogeneity analyses across the dimensions of economic development, gender, and age indicate that the implementation of the “Broadband China” policy has a stronger effect on obesity prevention in economically underdeveloped regions, among women, and among older adults. Analysis of the urban–rural dimension shows no significant intervention gap between urban and rural areas. On the other hand, an analysis of the educational dimension indicates that the policy has a more significant impact on the body weight of groups with higher levels of education. This suggests that differences in educational attainment may lead to a digital health divide, thereby posing challenges to the effectiveness of policy interventions.

**Table 7:** Results of the heterogeneity analysis.

Heterogeneity		BMI	Difference*	BMI-RD	Difference*
GDP	Developed (≥The mean of PGDP)	−0.169**	−0.149***	−0.003	−0.003***
		(0.075)		(0.002)	
	Underdeveloped (<The mean of PGDP)	−0.318***		−0.005***	
		(0.042)		(0.001)	
Urban and rural	Urban	−0.246***	−0.009	−0.001	0.004***
		(0.058)		(0.002)	
	Rural	−0.237***		−0.005***	
		(0.038)		(0.001)	
Gender	Men	−0.196***	−0.112***	−0.002*	−0.004***
		(0.044)		(0.001)	
	Women	−0.308***		−0.006***	
		(0.045)		(0.001)	
Age	Older adults (>65)	−0.371***	0.140***	−0.011***	0.008***
		(0.092)		(0.002)	
	Middle-aged and young adults (≤65)	−0.231***		−0.003***	
		(0.034)		(0.001)	
Edu	High level of education (years of education >9)	−0.265***	0.037*	−0.003*	−0.002*
		(0.056)		(0.001)	
	Low level of education (years of education ≤9)	−0.226***		−0.005***	
		(0.038)		(0.001)	

***, **, and * in the table denote significance levels at 1, 5, and 10%.

### Mechanism analysis

Table 8 presents the mechanism analysis results. Column (1) reports the effect of the “Broadband China” policy on mental health. The coefficient is −0.009 (*p* < 0.01), implying that the policy significantly lowers standardized psychological distress scores, with higher scores representing worse mental health conditions. This negative coefficient therefore signifies an improvement in mental health status: broadband infrastructure development alleviates residents’ psychological distress by expanding access to social support and emotional expression channels. Column (2) reports the effect on life satisfaction. The coefficient is 0.031 (*p* < 0.01), indicating that the policy significantly increases self-reported life satisfaction (measured on a 1–5 scale where higher values represent greater satisfaction). This suggests that digital infrastructure enhances residents’ subjective well-being by improving convenience of daily life, public service accessibility, and information acquisition. Column (3) displays the influence on daily working hours. The coefficient is-0.707 (*p* < 0.01), indicating the policy effectively cuts working hours, relieves time pressure and allows more time for healthy leisure activities. Column (4) shows the outcome for single exercise duration, with a coefficient of 0.227 (*p* < 0.01), proving the policy extends exercise time. It verifies that digital infrastructure promotes physical activity by reducing work burden and spreading fitness-related information. Column (5) reflects the effect on bedtime. The coefficient is 0.003 (*p* < 0.01), revealing that the policy delays bedtime, which may disturb biological rhythms and impede weight management. Column (6) shows the result for sedentary time. The coefficient is 0.207 (*p* < 0.01), suggesting the policy increases sedentary time, which squeezes out time for rest and physical exercise and is unfavorable to weight control.

**Table 8:** Mechanism analysis estimation results.

Variables	Mental health (1)	Life (2)	Work hour (3)	Exercise (4)	Sleep time (5)	Sedentariness (6)
Broadband	−0.009***	0.031***	−0.707***	0.227***	0.003***	0.207***
	(0.002)	(0.011)	(0.208)	(0.045)	(0.001)	(0.072)
Control variable	Yes	Yes	Yes	Yes	Yes	Yes
Constant	−0.028***	4.194***	−7.316***	1.717***	21.999***	13.275***
	(0.004)	(0.022)	(0.438)	(0.097)	(0.001)	(0.192)
City fixed effect	Yes	Yes	Yes	Yes	Yes	Yes
Time fixed effect	Yes	Yes	Yes	Yes	Yes	Yes
*R* ^2^	0.043	0.103	0.435	0.142	0.003	0.270
Observations	145,844	153,963	171,442	141,543	171,052	141,252

**, **, and * in the table denote significance levels at 1, 5, and 10%.

In summary, four influencing channels including relieved psychological distress, improved life satisfaction, reduced working hours and increased physical activity time constitute the core transmission paths through which digital infrastructure reduces obesity risk. By contrast, delayed sleep time and prolonged sedentary time serve as major negative competitive mechanisms that raise obesity risk. Hypotheses 2–4 are therefore validated.

## Conclusions and recommendations

Based on seven waves of CFPS data from 2010 to 2022, this study employs the difference-in-differences method to examine the impact of the “Broadband China” pilot policy on individual body weight. The study reaches the following main conclusions: First, the development of digital infrastructure significantly reduces obesity among individuals and effectively mitigates health inequalities. Second, heterogeneity analysis reveals a dual effect of the policy: on the one hand, the policy benefits disproportionately favor vulnerable groups such as those in underdeveloped regions, women, and older adults, effectively reducing the extent of health inequalities across various dimensions; on the other hand, differences in educational attainment may lead to unsatisfactory intervention effects of the policy on the body weight of low-skilled groups, exacerbating health inequality. Third, mechanism tests indicate that the development of digital infrastructure significantly reduces the incidence of obesity by mitigating the occurrence of mental health issues, enhancing perceived life satisfaction, reducing working hours, and increasing exercise time.

Based on the empirical findings of this study, we propose the following targeted policy recommendations:

First, accelerate inclusive digital infrastructure development. Our results demonstrate that the “Broadband China” policy significantly reduces obesity and health inequality, with stronger effects in economically underdeveloped regions. Policymakers should continue expanding high-quality broadband coverage to underserved areas by upgrading network infrastructure quality and reducing access costs, ensuring that the digital health dividend reaches all population segments equitably.

Second, bridge the digital health literacy gap through targeted interventions. Our heterogeneity analysis reveals that the policy’s body weight improvement effects are significantly weaker among populations with lower educational attainment (≤9 years of schooling), suggesting that educational disparities generate a “digital health literacy divide” that limits the effectiveness of infrastructure-level interventions. To address this, governments should: (a) organize accessible digital health literacy training programs specifically targeting residents in remote areas, older adults, and low-skilled workers; (b) simplify user interfaces of online medical services, health monitoring applications, and exercise guidance platforms; and (c) develop low-threshold digital health tools (e.g., voice-activated health reminders, simplified pictorial health information) to ensure policy benefits reach populations with limited digital competence.

Third, establish an inclusive monitoring and evaluation framework. Given the dual-edged nature of the policy’s distributional effects, we recommend that health authorities establish a systematic monitoring framework that tracks health outcome disparities across educational, age, and regional subgroups following digital infrastructure interventions. This framework should incorporate early-warning indicators for widening digital health divides and trigger complementary interventions (e.g., enhanced in-person outreach) when digital-only channels prove insufficient for specific populations.

Fourth, promote the deep integration of digital technology with public health services. Our mechanism analysis demonstrates that digital infrastructure operates through improving mental health, enhancing life satisfaction, reducing work time burdens, and increasing physical activity. Policymakers should leverage these pathways by supporting the development of digital mental health platforms, promoting flexible work policies enabled by broadband connectivity, and subsidizing online fitness content creation to translate technological capabilities into sustained population-level weight management outcomes.

## Data Availability

The datasets presented in this article are not readily available because this study uses data from the China Family Panel Studies (CFPS), a longitudinal survey conducted by the Institute of Social Science Survey (ISSS), Peking University. The CFPS public-use dataset is available to registered users through the official CFPS data platform (https://cfpsdata.pku.edu.cn/). City-level geographic identifiers used in this study are classified as restricted-access data by the CFPS and can only be accessed within the designated secure data enclave at the ISSS, Peking University, in accordance with the CFPS restricted data use agreement. Users are not permitted to publicly share CFPS data on journal websites or third-party platforms, either in its original or modified form. Researchers seeking to replicate the results may apply for access to the restricted data through the CFPS Data Management Office (email: cfpsdata@pku.edu.cn; website: https://www.isss.pku.edu.cn/cfps/) under the same conditions as the authors. The CFPS project was approved by the Peking University Biomedical Ethics Review Committee (IRB approval number: IRB00001052-14010), and informed consent was obtained from all participants or their guardians. The analytical code used in this study is provided as a Supporting Information file accompanying this article. Requests to access the datasets should be directed to cfpsdata@pku.edu.cn.
